# Adaptive Topographies and Equilibrium Selection in an Evolutionary Game

**DOI:** 10.1371/journal.pone.0116307

**Published:** 2015-02-23

**Authors:** Hinke M. Osinga, James A. R. Marshall

**Affiliations:** 1 Department of Mathematics, The University of Auckland, Private Bag 92019, Auckland 1142, New Zealand; 2 Department of Computer Science, University of Sheffield, Sheffield S1 4DP, United Kingdom; Peking University, CHINA

## Abstract

It has long been known in the field of population genetics that adaptive topographies, in which population equilibria maximise mean population fitness for a trait regardless of its genetic bases, do not exist. Whether one chooses to model selection acting on a single locus or multiple loci does matter. In evolutionary game theory, analysis of a simple and general game involving distinct roles for the two players has shown that whether strategies are modelled using a single ‘locus’ or one ‘locus’ for each role, the stable population equilibria are unchanged and correspond to the fitness-maximising evolutionary stable strategies of the game. This is curious given the aforementioned population genetical results on the importance of the genetic bases of traits. Here we present a dynamical systems analysis of the game with roles detailing how, while the stable equilibria in this game are unchanged by the number of ‘loci’ modelled, equilibrium selection may differ under the two modelling approaches.

## Introduction

Biological fitness typically depends on complicated phenotypes, which depend on multiple genetic loci. This raises an interesting modelling dilemma. At one extreme, one may model selection acting on phenotypes as if they were under simple genetic control at a single haploid locus; this is the ‘phenotypic gambit’ [1] widely used in evolutionary modelling, and referred to as evolutionary game theory when applied to model social interactions [2]. If multiple loci do underly a phenotype then, to make accurate evolutionary predictions, such models should capture inter-locus fitness interactions. Yet, they can be of much greater complexity, having to account for a number of phenotypes that may be exponential in the number of loci involved. At the other extreme, a very simple model may be formulated that considers selection acting independently on frequencies of different alleles at different loci. Such a model would be more tractable, but neglects important quantities such as linkage disequilibrium between loci. Hence, it may give incorrect predictions. An intermediate solution is also possible, through the adoption of multilocus population genetics [3–5].

In this paper, we examine the consequences of these two extreme approaches to modelling a simple general and classical problem: interactions in a social game where the players are assigned distinct roles [6]. Such interactions occur in many contexts, such as those where one individual possesses a territory and the other does not [6], interactions between adult reproductives and helpers [7], or between parents and offspring [8]. Even where payoffs are the same from both individuals’ perspectives, ‘uncorrelated asymmetries’ can lead to different behaviours being stable in the distinct roles, and these have previously been analysed in terms of evolutionary stable strategies at the strategy level [2, 6]. Recently, a new analysis of a social game with roles played between relatives has taken the independent gene-level view, and has shown that this gives the same attracting equilibria as the strategy-level view [9]; thus these equilibria correspond with the fitness-maximising evolutionary stable strategies of the game, regardless of whether they arise from a model using one locus or two. This is intriguing on several fronts. First, modelling selection at the strategy-level is akin to modelling selection acting on a larger number of genes competing for a single locus. Results from population genetics show that ‘adaptive topographies’ that take no account of the underlying genetic-basis of fitness do not exist; moving from modelling a trait using a single locus, to modelling that trait using multiple loci, can lead to population equilibria that do not correspond with population fitness maxima [10]. Second, the dimensions of the phase spaces of the two dynamical systems describing these different modelling levels are different, which means that one should not expect their behaviour to be the same. We show in this paper that a projection of the higher-dimensional system onto the phase space of the other still does not lead to a topologically equivalent system. We present a dynamical systems analysis of both systems in order to elucidate their differences. In particular, we show that they do not have equal numbers of equilibria, but for both models there are always at most two stable coexisting equilibria, and the same stable equilibria exist in both models under the same parametrisations. Despite their identical stable equilibria, equilibrium selection behaviour in the two can differ; seemingly equivalent initial conditions in the two systems can lead them to converge to different stable equilibria.

## Analysis

### Donation games with roles played between relatives

We consider the donation game with potentially non-additive payoffs as presented in Table 1. Interactions are structured such that there is an ‘uncorrelated asymmetry’ [2]; that is, players occupy distinct behavioural roles, and have different strategies according to the role they occupy. Interactions are further structured such that they occur between genetic relatives [9]. This form of the game provides insight into a particular problem of biological interest, namely selection of non-additive social behaviours between relatives; however, the payoff matrix is equivalent to the original fully general payoff matrix [2], as shown in [9], and hence could capture other biologically-interesting interactions. If we wish to model changes in frequencies, rather than changes in value of a trait that the population is monomorphic for, as in adaptive dynamics [11], then there are two different ways of modelling such a game as a dynamical system. On the one hand, the dynamical system can describe the evolution of the frequencies of all possible strategies, which we shall label genotypes. The set of all possible genotypes for the donation game, denoted G, contains four elements, namely, G={CC,CD,DC,DD}. The genotype dynamics is modelled by the rates of change of the frequencies *f*_•_ of individuals with these four genotypes, with •∈G. The equations are of the form

$$\begin{array}{c} {\dot{f}}_{•}=f_{•}\left(w_{•}-\bar{w}\right),\;•\in G. \end{array}$$ (1)

Here, *w*_•_ is the inclusive fitness of an individual with a given genotype and $\bar{w}$ is the population mean fitness defined as

$$\begin{array}{c} \bar{w}=f_{\mathrm{CC}}w_{\mathrm{CC}}+f_{\mathrm{CD}}w_{\mathrm{CD}}+f_{\mathrm{DC}}w_{\mathrm{DC}}+f_{\mathrm{DD}}w_{\mathrm{DD}}. \end{array}$$

The inclusive fitnesses of individuals with the different genotypes are given in the equivalent neighbour-modulated fitness form [12] by

$$\begin{array}{ccc} w_{\mathrm{CC}} & = & r(b+d)-c+\\ & & \left(1-r\right)\left[f_{\mathrm{CC}}+\frac{1}{2}f_{\mathrm{CD}}+\frac{1}{2}f_{\mathrm{DC}}\right]\;\left(b+d\right), \end{array}$$ (2)

$$\begin{array}{ccc} w_{\mathrm{CD}} & = & \frac{1}{2}\left(rb-c\right)+\\ & & \frac{1}{2}\left(1-r\right)\left[f_{CC}\left(2b+d\right)+f_{\mathrm{CD}}b+f_{\mathrm{DC}}\left(b+d\right)\right], \end{array}$$ (3)

$$\begin{array}{ccc} w_{\mathrm{DC}} & = & \frac{1}{2}\left(rb-c\right)+\\ & & \frac{1}{2}\left(1-r\right)\left[f_{\mathrm{CC}}\left(2b+d\right)+f_{\mathrm{CD}}\left(b+d\right)+f_{\mathrm{DC}}b\right], \end{array}$$ (4)

$$\begin{array}{ccc} w_{\mathrm{DD}} & = & \left(1-r\right)\left[f_{\mathrm{CC}}+\frac{1}{2}f_{\mathrm{CD}}+\frac{1}{2}f_{\mathrm{DC}}\right]b. \end{array}$$ (5)

Here, 0 ≤ *r* ≤ 1 is the average degree of relatedness between interacting individuals within the population, giving the probability that interactants have identical genotypes over-and-above that given by the population frequencies of individuals with these genotypes [13]. In the formulation above, we used the fact that the sum of the frequencies is 1, that is, *f*_DD_ = 1 − (*f*_CC_ + *f*_CD_ + *f*_DC_).

**Table 1:** Payoffs for the non-additive donation game.

	**C**	**D**
**C**	*b* − *c* + *d*	−*c*
**D**	*b*	0

As discussed in [9], the simplest alternative model is based on selection acting independently on the frequencies of sub-strategies for each behavioural role. Here, one describes the rate of change in the frequency of each allele for each role; we shall sometimes refer to this as the gene dynamics view. There are two roles (players) in the donation game and two different alleles, namely, cooperative C and defective D. Since the frequencies in both roles again add up to 1, we only consider the frequencies *ϕ*_C1_ and *ϕ*_C2_ of cooperative alleles occuring in each of the two roles; the frequencies of defective alleles, and corresponding equations, follow from the equalities *ϕ*_D1_ = 1 − *ϕ*_C1_ and *ϕ*_D2_ = 1 − *ϕ*_C2_. The replicator dynamics [14] is then defined as

$$\begin{array}{c} {\dot{\phi }}_{Ci}=\phi_{Ci}\left(1-\phi_{Ci}\right)\left(\omega_{Ci}-\omega_{Di}\right), \end{array}$$ (6)

where *i*, *j* ∈ {1, 2} and *i* ≠ *j*. The inclusive fitnesses of cooperative and defective alleles for each case are now given by

$$\begin{array}{ccc} \omega_{C1} & = & -c+\left(b+d\right)\phi_{C2}+r\left[b+\left(-c+d\right)\phi_{C2}\right], \end{array}$$ (7)

$$\begin{array}{ccc} \omega_{D1} & = & b\phi_{C2}-rc\phi_{C2}, \end{array}$$ (8)

$$\begin{array}{ccc} \omega_{C2} & = & -c+\left(b+d\right)\phi_{C1}+r\left[b+\left(-c+d\right)\phi_{C1}\right], \end{array}$$ (9)

$$\begin{array}{ccc} \omega_{D2} & = & b\phi_{C1}-rc\phi_{C1}. \end{array}$$ (10)

Here, *ω*_C1_, *ω*_D1_, *ω*_C2_ and *ω*_D2_ are defined in terms of the two distintinct behavioural contexts, which are mutually exclusive [9]. For example, *ω*_C1_ is the inclusive fitness of a cooperation allele specifying behaviour for role 1; the first two terms give the expected direct pay-off to the cooperative allele 1 arising from the behaviour it encodes (cooperation), depending on whether allele 2 is of type C or D, while the last term gives the expected pay-off for allele 2 in the social partner, weighted by the relatedness of that partner to the focal individual who occupies role 1.

Formulations (1) and (6) both describe the evolution of four different frequencies, but the dynamical systems are not the same. In particular, note that the state of system (1) is determined by three of the four frequencies, since *f*_CC_ + *f*_CD_ + *f*_DC_ + *f*_DD_ = 1; this means that the phase space of system (1) has dimension three. The state of system (6), however, is already determined by two of the four frequencies, since *ϕ*_C1_ + *ϕ*_D1_ = 1 and *ϕ*_C2_ + *ϕ*_D2_ = 1, and the dimension of its phase space is only two. Due to this difference in dimensions, the two systems cannot be topologically equivalent [15, 16] and one should expect that the behaviour of the two systems is not the same. One may be tempted to believe that the higher-dimensional system (1) implies the behaviour of system (6), because *ϕ*_C1_ and *ϕ*_C2_ should evolve in the same way as *f*_CC_ + *f*_CD_ and *f*_CC_ + *f*_DC_, respectively. However, it is not hard to show that also in this sense the two systems are not topologically equivalent. While the proof is straightforward, it is rather tedious and not very insightful. Therefore, we provide this proof in Supporting Information (S1 Appendix).

Despite this lack of topological equivalence between systems (1) and (6), it might be assumed that the two systems have the same number of stable equilibria and predictions of the long-term behaviour made using either model give the same results. In this paper, we explain in detail that systems (1) and (6) can, in fact, give conflicting predictions for the long-term behaviour. We discuss the equilibria and stability properties for system (1) and compare predictions from system (1) with predictions from system (6) by defining *ϕ*_C1_ = *f*_CC_ + *f*_CD_ and *ϕ*_C2_ = *f*_CC_ + *f*_DC_. In the following section, we first consider system (6).

### Analysis of equilibrium states for the gene dynamics model

A detailed analysis of the equilibria for system (6) in their most general form has already been provided in [9]. We present here the analysis as is standard in dynamical systems theory [15, 17] and determine stability properties using the Jacobian matrix; this same approach will also be used for the analysis of system (1). The two-dimensional system (6) can be rewritten explicitly in terms of the two variables *ϕ*_C1_ and *ϕ*_C2_ and the parameters *b*, *c*, *d* and *r* as

$$\begin{array}{c} \left\{\begin{array}{ccc} {\dot{\phi }}_{C1} & = & \phi_{C1}\left(1-\phi_{C1}\right)\left(rb-c+\left[1+r\right]d\phi_{C2}\right),\\ {\dot{\phi }}_{C2} & = & \phi_{C2}\left(1-\phi_{C2}\right)\left(rb-c+\left[1+r\right]d\phi_{C1}\right). \end{array}\right. \end{array}$$

Recall that the dynamics for *ϕ*_D1_ and *ϕ*_D2_ can readily be deduced from the relationships *ϕ*_D1_ = 1 − *ϕ*_C1_ and *ϕ*_D2_ = 1 − *ϕ*_C2_. We focus here on the cases *d* > 0 and *d* < 0, where we assume *b*, *c* > 0 and 0 < *r* < 1.

Equilibria are found as solutions that simultaneously satisfy ${\dot{\phi }}_{\text{C}1}=0$ and ${\dot{\phi }}_{\text{C}2}=0$. The equality ${\dot{\phi }}_{\text{C}1}=0$ holds if

$$\begin{array}{ccc} & & \phi_{C1}=0,\;\phi_{C1}=1,\\ & \text{or} & \phi_{C2}=\frac{c-rb}{(1+r)d}:=e^{*}. \end{array}$$

Similarly, the equation ${\dot{\phi }}_{\text{C}2}=0$ is satisfied if *ϕ*_C2_ = 0, *ϕ*_C2_ = 1 or *ϕ*_C1_ = e*. Hence, system (6) always has the four equilibria (*ϕ*_C1_, *ϕ*_C2_) = (0, 0), (1, 0), (0, 1) and (1, 1), and there exists a fifth equilibrium

$$\begin{array}{c} \left(\phi_{C1},\phi_{C2}\right)=\left(e^{*},e^{*}\right), \end{array}$$

provided 0 < e* < 1, that is,

$$\begin{array}{ccc} & & 0<\frac{c-rb}{(1+r)d}<1\Leftrightarrow \\ & & \left\{\begin{array}{ccccccc} d>0 & \text{and} & \frac{c-d}{b+d} & < & r & < & \frac{c}{b},\\ d<0 & \text{and} & \frac{c}{b} & < & r & < & \frac{c-d}{b+d}, \end{array}\right. \end{array}$$

provided *b* + *d* > 0. Here, we used the assumptions *b* > 0 and 0 < *r* < 1 made earlier in this section; note that *b* + *d* > 0 amounts to assuming that any negative non-additive payoffs *d*, arising when two donators interact, are not large enough to offset the benefits *b* arising from donation. The conditions on the right-hand side of the double-implication above correspond to conditions A.4 and A.5 derived in [9].

The stability of these equilibria is determined by the eigenvalues of the Jacobian matrix, obtained from linearizing system (6) about the respective equilibria. Let us define

$$\begin{array}{c} e\left(\phi \right):=\left(rb-c+[1+r]d\phi \right). \end{array}$$

The Jacobian matrix at an equilibrium (*ϕ*_C1_, *ϕ*_C2_) is then defined as

$$\begin{array}{ccc} & & \mathrm{Jac}(\phi_{C1},\phi_{C2})=\\ & & \left(\begin{array}{cc} \left(1-2\phi_{C1}\right)e\left(\phi_{C2}\right) & \phi_{C1}\left(1-\phi_{C1}\right)e^{'}\left(\phi_{C2}\right)\\ \phi_{C2}\left(1-\phi_{C2}\right)e^{'}\left(\phi_{C1}\right) & \left(1-2\phi_{C2}\right)e\left(\phi_{C1}\right) \end{array}\right). \end{array}$$

Hence, the Jacobian matrices for (*ϕ*_C1_, *ϕ*_C2_) = (0, 0) and (*ϕ*_C1_, *ϕ*_C2_) = (1, 1) are diagonal matrices with double eigenvalues *e*(0) = *rb* − *c* and −*e*(1) = *c* − *d* − *r*(*b* + *d*), respectively. Therefore, the origin is stable if and only if *e*(0) < 0 ⇔ *r* < *c*/*b*. If we again assume *b* + *d* > 0, then (1, 1) is stable if and only if −*e*(1) < 0 ⇔ *r* > (*c* − *d*)/(*b* + *d*). We conclude that (0, 0) and (1, 1) are both stable precisely when (e*, e*) exists. This equilibrium (*ϕ*_C1_, *ϕ*_C2_) = (e*, e*), has the anti-diagonal Jacobian matrix

$$\begin{array}{ccc} & & \mathrm{Jac}(1,0)=\\ & & \left(\begin{array}{cc} 0 & e^{*}\left[1-e^{*}\right]\left(1+r\right)d\\ e^{*}\left[1-e^{*}\right]\left(1+r\right)d & 0 \end{array}\right), \end{array}$$

with eigenvalues ±e*[1 − e*](1 + *r*)*d*. Hence, (e*, e*) is always a saddle equilibrium. Finally, the Jacobian matrices for (*ϕ*_C1_, *ϕ*_C2_) = (1, 0) and (*ϕ*_C1_, *ϕ*_C2_) = (0, 1) are diagonal matrices with both the same eigenvalues, namely, −*e*(0) = *c* − *rb* and *e*(1) = *r*(*b* + *d*) − (*c* − *d*). Therefore, (1, 0) and (0, 1) are sources in the parameter regime where (0, 0) and (1, 1) are both stable. Otherwise, they are typically saddles, because stability of (1, 0) and (0, 1) requires (*c* − *d*)/(*b* + *d*) > *c*/*b* and this can only hold if *d* < 0; see also [9].

**Fig 1:**
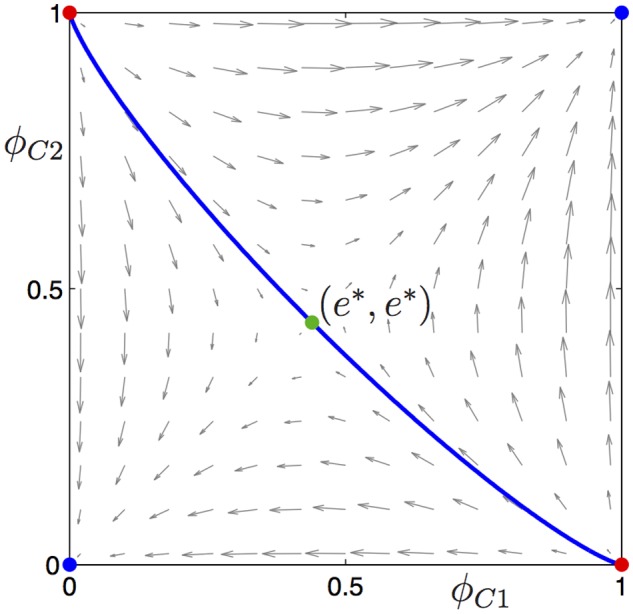
Phase portrait illustrating bistability of the equilibria (0, 0) and (1, 1) for the gene system (6) with parameters *b* = 2, *c* = 0.5, *d* = 0.25 > 0, and *r* = 0.185. The (solid blue) curve through the saddle equilibrium (e*, e*) ≈ (0.4388, 0.4388) is the stable manifold of (e*, e*) that separates the two basins of attraction for (0, 0) and (1, 1).

To illustrate the global behaviour of the gene dynamics model (1), let us consider an example of a situation where the equilibrium (e*, e*) exists; as parameters, we choose *b* = 2, *c* = 0.5, *d* = 0.25 > 0, and *r* = 0.185. Fig. 1 shows the phase portrait for this case in the (*ϕ*_*C*1_, *ϕ*_*C*2_)-plane. The (gray) arrows indicate the direction of the flow and clearly show a situation of bistability, with both (0, 0) and (1, 1) (blue dots) attracting nearby points. Note that (1, 0) and (0, 1) (red dots) are both sources, because nearby points all move away from these two equilibria. The basins of the two attracting equilibria are separated by two trajectories of points that flow from the respective two sources to the saddle equilibrium (e*, e*) ≈ (0.4388, 0.4388); all other points near (e*, e*) flow away from (e*, e*). These two special trajectories form the stable manifold of (e*, e*) that acts as a separatrix for the two attractors at (0, 0) and (1, 1).

### Behaviour of the gene dynamics model as *r* varies from 0 to 1

**Fig 2:**
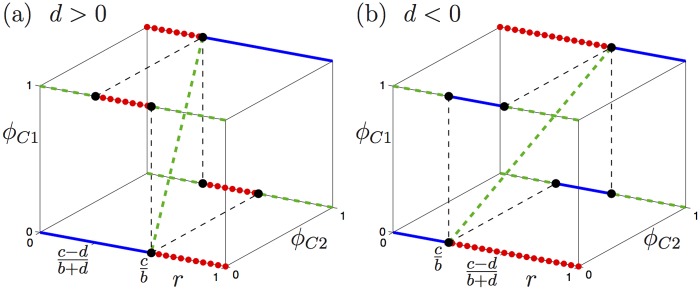
Bifurcation diagrams with 0 < *r* < 1 of the gene model with *d* > 0 (a) and *d* < 0 (b); here, we asssume *c* > *b* > 0 are such that 0 < (*c* − *d*)/(*b* + *d*) < 1 (which is automatically satisfied if *d* > 0, but not if *d* < 0). The stability of the equilibria is indicated by solid (blue), dashed (green) and dotted (red) lines for attractors, saddles and repellors, respectively.

We are primarily interested in how the stability of the equilibrium states change as the degree *r* of relatedness varies between 0 and 1. We again refer to the results in [9] for comparison. The two cases *d* > 0 and *d* < 0 are different and we first consider the case *d* > 0. If *d* > 0 then (*c* − *d*)/(*b* + *d*) < *c*/*b*. The existence and stability properties of the equilibria are illustrated in Fig. 2(a), where we plot the equilibria in the (*ϕ*_C1_, *ϕ*_C2_)-plane versus *r*. For *r* < (*c* − *d*)/(*b* + *d*), the origin is a global attractor (solid blue line), (1, 1) is a repellor (dotted red line), (1, 0) and (0, 1) are saddles (dashed green lines), and (e*, e*) does not exist. When *r* = (*c* − *d*)/(*b* + *d*) a bifurcation occurs and the equilibrium (e*, e*) emerges from the (1, 1)-branch; this bifurcation is a transcritical bifurcation, but it is degenerate due to the symmetries of the model and not only the stability of (1, 1), but also of (1, 0) and (0, 1) changes. For (*c* − *d*)/(*b* + *d*) < *r* < *c*/*b*, both the origin and (1, 1) are attractors (solid blue lines), (1, 0) and (0, 1) are repellors (dotted red lines), and (e*, e*) is a saddle (dashed green line); as illustrated in Fig. 1, the stable manifold of (e*, e*) separates the basins of the two attractors in the system. At *r* = *c*/*b*, another transcritical bifurcation occurs, which is similarly degenerate, where (e*, e*) merges with the origin and again (1, 0) and (0, 1) change stability as well. For *r* > *c*/*b*, the origin is a repellor (dotted red line), (1, 1) is now the global attractor (solid blue line), (1, 0) and (0, 1) are again saddles (dashed green lines), and (e*, e*) no longer exists.

The situation for *d* < 0 is quite different for the parameter regime where (e*, e*) exists, because it gives rise to bistability of the off-diagonal equilibria (1, 0) and (0, 1). The corresponding bifurcation diagram is shown in Fig. 2(b). The equilibrium (e*, e*) can only exist if *b* and *c* are such that 0 < *c*/*b* < (*c* − *d*)/(*b* + *d*) < 1. As before, the origin is the global attractor as long as *r* < *c*/*b*; the equilibrium (1, 1) is a repellor, (1, 0) and (0, 1) are saddles, and (e*, e*) does not exist. There are again two (degenerate) transcritical bifurcations, one at *r* = *c*/*b* and one at *r* = (*c* − *d*)/(*b* + *d*), where the equilibrium (e*, e*) merges in opposite order with (0, 0) and (1, 1), respectively. This means that both the origin and (1, 1) are repellors when *c*/*b* < *r* < (*c* − *d*)/(*b* + *d*), and the equilibria (1, 0) and (0, 1) are the attractors. In this regime, (e*, e*) is again a saddle and its stable manifold separates the basins of (1, 0) and (0, 1); one branch of the stable manifold of (e*, e*) connects to (0, 0) and the other to (1, 1). As for the case *d* > 0, if *r* > (*c* − *d*)/(*b* + *d*) then the origin is a repellor, (1, 1) is the global attractor and (1, 0) and (0, 1) are saddles; the equilibrium (e*, e*) no longer exists.

Let us mention here that the equilibrium (e*, e*) only occurs if *d* ≠ 0. If *d* = 0 then (1, 0) and (0, 1) are always saddles, and the origin is the global attractor for *r* < *c*/*b*, with (1, 1) a repellor, while it is a repellor for *r* > *c*/*b*, when (1, 1) is the global attractor. The bifurcation at *r* = *c*/*b* is highly degenerate in this case.

**Remark 1** *The local stability analysis, along with the location of the stable manifolds of the saddles, completely determines the global behaviour of the system. In particular, the only attractors for system* (6) *are equilibria, which follows from the Poincaré-Bendixson theorem for planar systems: Any other attractor must be a periodic orbit. Since the lines* {*ϕ*_C1_ = 0}, {*ϕ*_C1_ = 1}, {*ϕ*_C2_ = 0}, *and* {*ϕ*_C2_ = 1} *are all invariant for system* (6), *the only possible rotation must occur around an equilibrium in the interior of the square* [0, 1] × [0, 1]; *however*, (e*, e*) *is always a saddle, which prevents the existence of a surrounding periodic orbit. See also* [16, 18].

### Analysis of equilibrium states for the genotype model

As we already mentioned, it might be assumed that stable equilibria of the gene dynamics model (6) should correspond to stable equilibria of the genotype model (1) and, more importantly, in the case of bistability, both systems should have the same predictions for corresponding initial conditions. Therefore, we now compare our findings for the gene dynamics model with a similar equilibrium analysis for the genotype model (1). Recall that the genotype dynamics is modelled as

$$\begin{array}{c} {\dot{f}}_{•}=f_{•}\left(w_{•}-\bar{w}\right), \end{array}$$

with •∈G={CC,CD,DC,DD}. In its most general form, this system is fully determined by the dynamics of the frequencies *f*_CC_, *f*_CD_ and *f*_DC_, with *f*_DD_ given by the relationship *f*_CC_ + *f*_CD_ + *f*_DC_ + *f*_DD_ = 1. Its equilibria satisfy

$$\begin{array}{c} f_{•}=0\;\text{or}\;w_{•}=\bar{w} \end{array}$$ (11)

for all combinations •∈G. Note that we cannot have all *f*_•_ = 0, or in other words, the origin (0, 0, 0, 0) is not a solution, because we require *f*_CC_ + *f*_CD_ + *f*_DC_ + *f*_DD_ = 1. We can show that there also exist no equilibria with all *f*_•_ ≠ 0 and we have the following Lemma.

**Lemma 1** *If* (*f*_CC_, *f*_CD_, *f*_DC_, *f*_DD_) *is an equilibrium of* (1) *with* 0 < *r* < 1 *and* *d* ≠ 0 *then*

$$\begin{array}{c} f_{\mathrm{CC}}\cdot f_{\mathrm{CD}}\cdot f_{\mathrm{DC}}\cdot f_{\mathrm{DD}}=0, \end{array}$$

*that is, at least one of its coordinates is zero*.

The proof of Lemma 1 is given at the end of the Analysis section.

Equation (11) provides a systematic way to derive all possible equilibria of (1). Furthermore, we can use the number of nonzero coordinates as a guide to ensure we find all of them. This leads to the following complete classification of equilibria of (1).

**Theorem 2** *Consider the genotype dynamics modelled as system* (1) *with* *d* ≠ 0 *and* 0 < *r* < 1. *There are at most eight equilibria, of which at most seven can coexist for a small range of r depending on the choice of the parameters b, c* > 0. *Based on their numbers of nonzero coordinates, we distinguish three classes:*

(i) *There are four equilibria with a single nonzero coordinate. These are* (1, 0, 0, 0), (0, 1, 0, 0), (0, 0, 1, 0), *and* (0, 0, 0, 1), *which exist for all* 0 < *r* < 1.
(ii) *There are two equilibria with two nonzero coordinates, namely*, $\left(0,\frac{1}{2},\frac{1}{2},0\right)$, *which exists for all* 0 < *r* < 1, *and*
  $$\begin{array}{c} E_{23}:=\left(\frac{c-r(b+d)}{(1-r)d},0,0,\frac{d-c+rb}{(1-r)d}\right). \end{array}$$ (12)
  *The equilibrium* *E*_23_ *only exists if* *c* − *b* < *d* (*for either* *d* > 0 *or* *d* < 0), *and its bounds of existence are defined by*
  $$\begin{array}{c} r_{23}^{b}:=\max\left(0,\frac{c-d}{b}\right)<1, \end{array}$$
  *and*
  $$\begin{array}{c} r_{23}^{e}:=\frac{c}{b+d}\in \left(0,1\right). \end{array}$$
  *If* *d* > 0 *then* *E*_23_ *exists for* $r_{23}^{b}<r<r_{23}^{e}$; *this range becomes* $r_{23}^{e}<r<r_{23}^{b}$ *if* *d* < 0.
(iii) *There are also two equilibria with three nonzero coordinates, but these must have* *f*_CD_ = *f*_DC_; *here, either* *f*_CC_ = 0 *or* *f*_DD_ = 0. *The first equilibrium is*
  $$\begin{array}{c} E_{1}:=\left(0,\frac{c-rb}{(1-r)d},\frac{c-rb}{(1-r)d},\frac{d-2c+r(2b-d)}{(1-r)d}\right). \end{array}$$ (13)
  *If we assume* 2*b* − *d* > 0, *then* *E*_1_ *can only exist if* *b* > *c* *and its bounds of existence become*
  $$\begin{array}{c} r_{1}^{b}:=\max\left(0,\frac{2c-d}{2b-d}\right)<1, \end{array}$$
  *and*
  $$\begin{array}{c} r_{1}^{e}:=\frac{c}{b}<1. \end{array}$$
  *As before*, *E*_1_ *exists only for* $r_{1}^{b}<r<r_{1}^{e}$ *if* *d* > 0 *and only for* $r_{1}^{e}<r<r_{1}^{b}$ *if* *d* < 0. *For the case* 2*b* − *d* < 0, *we must have* *d* > 0 *and* *E*_1_ *can exist only if* $c<\frac{1}{2}d$, *with bounds* $0<r<r_{1}^{e}<1$ *if* *b* > *c* *and* $0<r<r_{1}^{b}<1$ *if* *b* < *c*. *The only other possible equilibrium is*
  $$\begin{array}{c} \begin{array}{c} E_{4}:=\left(\frac{2c-d-r(2b+3d)}{(1-r)d},\frac{d-c+r(b+d)}{(1-r)d},\right.\;\;\;\;\\ \left.\frac{d-c+r(b+d)}{(1-r)d},0\right). \end{array} \end{array}$$ (14)
  *Existence of* *E*_4_ *requires* $\frac{1}{2}(c-b)<d$ *and the bounds on r become*
  $$\begin{array}{c} r_{4}^{b}:=\max\left(0,\frac{c-d}{b+d}\right)<1, \end{array}$$
  *and*
  $$\begin{array}{c} r_{4}^{e}:=\max\left(0,\frac{2c-d}{2b+3d}\right)<1. \end{array}$$
  *Again, if* *d* > 0 *then* *E*_4_ *exists only for* $r_{4}^{b}<r<r_{4}^{e}$ *and the range becomes* $r_{4}^{e}<r<r_{4}^{b}$ *if* *d* < 0.

The proof of Theorem 2 is given at the end of the Analysis section.

### Contradicting predictions from the gene dynamics and genotype models

**Fig 3:**
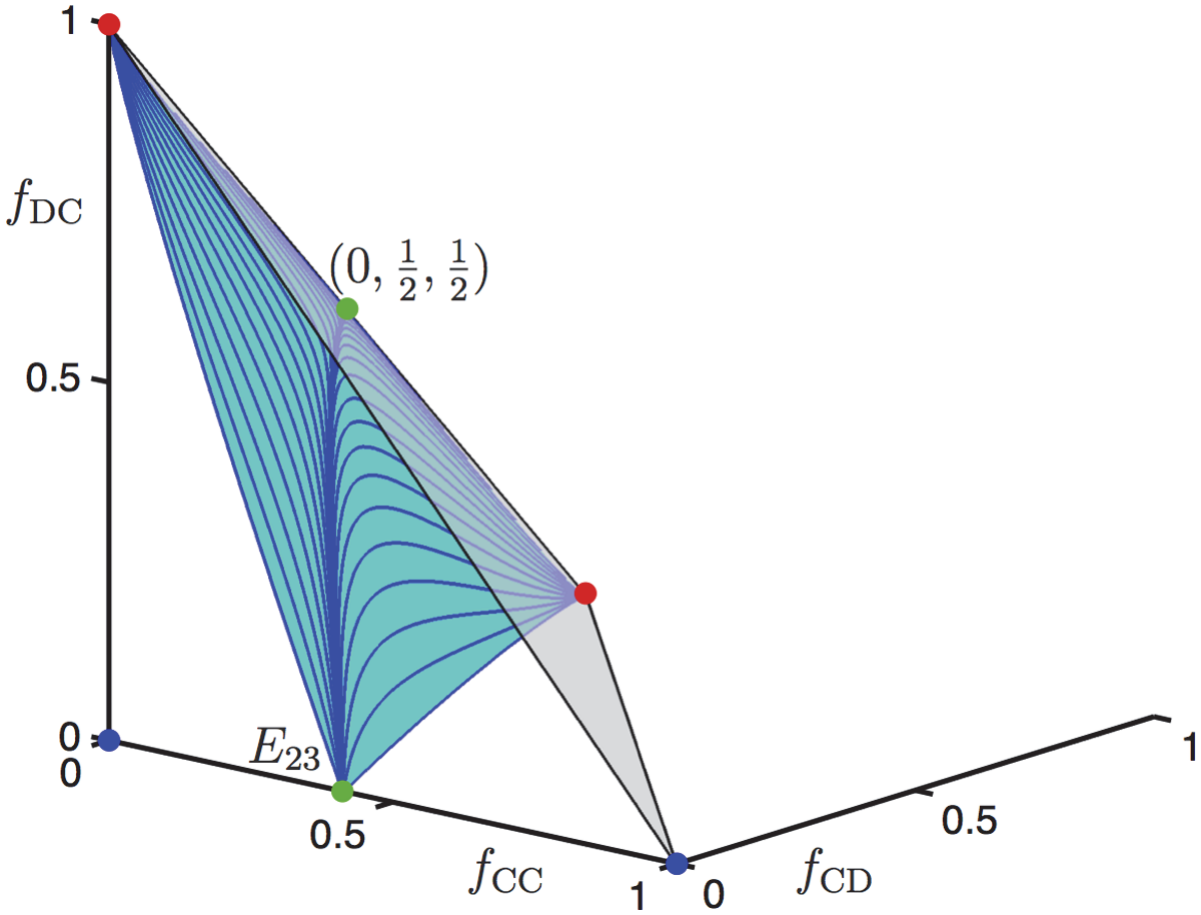
Phase portrait in (*f*_CC_, *f*_CD_), *f*_DC_)-space illustrating bistability of the equilibria (0, 0, 0) and (1, 0, 0) for the genotype model (1) with parameters *b* = 2, *c* = 0.5, *d* = 0.25 > 0, and *r* = 0.185. The (blue) surface emanating from the saddle equilibrium *E*_23_ ≈ (0.4110, 0, 0) is the stable manifold of *E*_23_ that separates the two basins of attraction for (0, 0, 0) and (1, 0, 0); compare also Fig. 1.

As an illustration of the global behaviour of the genotype model (1), let us consider the same parameter values used for the gene dynamics model (6) in Fig. 1, namely, *b* = 2, *c* = 0.5, *d* = 0.25 > 0, and *r* = 0.185. The gene dynamics model (6) has five equilibria for this choice of parameters, which is the largest possible number of equilibria for this two-dimensional model. For the genotype model (1) we find six co-existing equilibria. This model is three dimensional and the (*f*_CC_, *f*_CD_, *f*_DC_)-coordinates of these equilibria are (0, 0, 0), (1, 0, 0), (0, 1, 0), (0, 0, 1), along with $\left(0,\frac{1}{2},\frac{1}{2}\right)$ and *E*_23_ ≈ (0.4110, 0, 0). A phase portrait is shown in Fig. 3. Note that the phase space is confined to the tetrahedron bounded by the three coordinate planes *f*_CC_ = 0, *f*_CD_ = 0, *f*_DC_ = 0, and the plane *f*_CC_ + *f*_CD_ + *f*_DC_ = 1. We find that two of the equilibria are stable, namely, (0, 0, 0) and (1, 0, 0). The equilibria (0, 1, 0) and (0, 0, 1) are sources, and $\left(0,\frac{1}{2},\frac{1}{2}\right)$ and *E*_23_ are saddles. The equilibrium *E*_23_ is the only equlibrium with a two-dimensional stable manifold and it is this surface that separates the basins of the two attracting equilibria in phase space. We computed the stable manifold of *E*_23_ by continuation of a two-point boundary value problem with the package Auto [19, 20]; the formulation of this computational method is described in [21, 22]. Our computation shows that the stable manifold of *E*_23_ is an almost planar surface. It intersects the tetrahedron that defines the phase space of the gene dynamics model (6) in two curves along the sides *f*_*CD*_ = 0 and *f*_*DC*_ = 0, and the closure of this two-dimensional stable manifold includes the straight line *f*_*CD*_ + *f*_*DC*_ = 1 on the side *f*_*CC*_ = 0.

**Fig 4:**
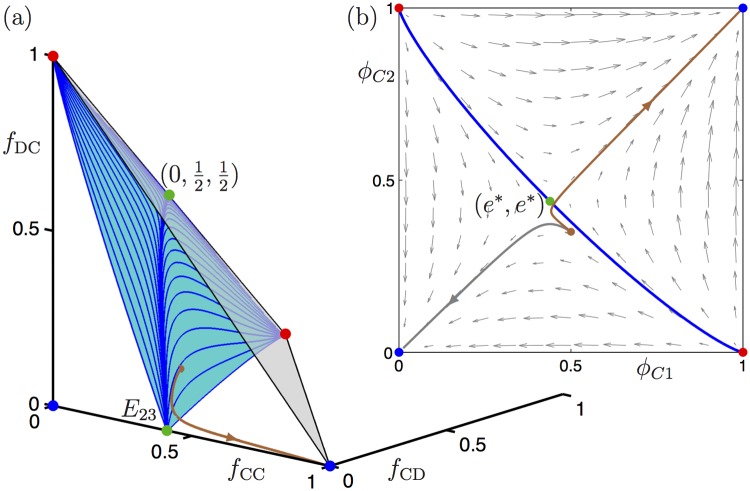
The initial conditions for a trajectory of the genotype model (1) that converges to the attracting equilibrium (1, 0, 0) in (*f*_CC_, *f*_CD_, *f*_DC_)-space can project onto the two-dimensional phase plane *f*_C1_ = *f*_CC_ + *f*_CD_ and *f*_C2_ = *f*_CC_ + *f*_DC_ such that the corresponding trajectory for the gene dynamics model (6) converges to the equilibrium (0, 0). Panel (a) shows the trajectory for (1) in (*f*_CC_, *f*_CD_, *f*_DC_)-space (brown curve) and panel (b) shows the corresponding projection overlayed on the phase portrait for the gene dynamics model (6); the expected trajectory as defined by (6) is shown in grey.

Despite the fact that there are more equilibria than for the gene dynamics model (6), the phase portrait of the genotype model (1) in Fig. 3 seems rather similar: comparing Fig. 1, there are two attracting equilibria separated by the stable manifold of a saddle equilibrium; since the equilibrium $\left(0,\frac{1}{2},\frac{1}{2}\right)$ of the genotype dynamics model (1) is contained in the closure of the separatrix, its role in the global dynamics is determined by the stable manifold of *E*_23_. Furthermore, just as for (e*, e*) in model (6), the equilibrium *E*_23_ lies roughly in the middle between the two attracting equilibria. In order to compare the dynamics of these two systems (1) and (6) in more detail, we define the variables *f*_C1_: = *f*_CC_ + *f*_CD_ and *f*_C2_: = *f*_CC_ + *f*_DC_ as given by system (1). The two systems could be considered equivalent if any trajectory for system (1) would give rise to a projection onto (*f*_C1_, *f*_C2_)-coordinates that maps one-to-one to a trajectory for system (6). Note that there is a one-to-one correspondence between the equilibria (0, 0, 0), (1, 0, 0), (0, 1, 0) and (0, 0, 1) of (1) in class (i) and the equilibria (0, 0), (1, 0), (0, 1) and (1, 1) of (6). However, none of the equilibria of (1) map to the equilibrium (e*, e*) of (6). This can have dramatic consequences for the behaviour of the two systems. In particular, it should be possible to choose an initial condition in (*f*_CC_, *f*_CD_, *f*_DC_)-space that lies in the basin of (1, 0, 0), that is, to the right of the stable manifold of *E*_23_, such that its projection onto the (*f*_C1_, *f*_C2_)-plane lies in the basin of (0, 0). An example to this effect is given in Fig. 4. Here, we consider again the parameters *b* = 2, *c* = 0.5, *d* = 0.25 > 0, and *r* = 0.185, and choose the initial condition (*f*_CC_, *f*_CD_, *f*_DC_) = (0.25, 0.25, 0.1). Under the flow of (1), this point converges to (1, 0, 0), as indicated by the (brown) curve in Fig. 4(a). However, the projection onto the (*f*_C1_, *f*_C2_)-plane of this trajectory starts from the initial condition (0.5, 0.35), which lies in the basin of (0, 0) with respect to the flow of (6); the projected trajectory (brown curve) and the trajectory as dictated by (6) (grey curve) are shown in Fig. 4(b).

The example discussed above is a numerical illustration of the following important conjecture.

**Proposition 3** *Suppose the parameters* *b*, *c* > 0, *d* ≠ 0 *and* 0 < *r* < 1 *are chosen such that the gene dynamics model* (6) *exhibits bistability between attracting equilibria* *A*_1_ *and* *A*_2_. *Consider the basin of attraction of* *A*_1_, *denoted* $B(A_{1})$, *and an initial condition* $\left(\phi_{\text{C}1},\phi_{\text{C}2})\in B(A_{1}\right)$, *that is, the trajectory through* (*ϕ*_C1_, *ϕ*_C2_) *converges to* *A*_1_. *Let* (*f*_CC_, *f*_CD_, *f*_DC_) *be an initial condition of the genotype system* (1) *with* *f*_CC_ + *f*_CD_ = *ϕ*_C1_ *and* *f*_CC_ + *f*_DC_ = *ϕ*_C2_; *here, we use the same values for* *b*, *c*, *d* *and* *r* *as for* (6). *It is possible to choose* (*f*_CC_, *f*_CD_, *f*_DC_) *such that* $\left(\phi_{\text{C}1},\phi_{\text{C}2})\in B(A_{1}\right)$, *but the trajectory associated with the flow of* (1) *does not converge to an attractor that corresponds to* *A*_1_.

Proposition 3 is motivated by the fact that there is no candidate equilibrium of (1) that corresponds to the equilibrium (e*, e*) of (6). This equilibrium is important, because its stable manifold acts as a separatrix between the basins of *A*_1_ and *A*_2_. If we assume that the genotype system (1) also exhibits bistability for the chosen parameter values, then there must exist an equilibrium of (1) and corresponding stable manifold that acts as a separatrix in a similar way. The projection of this stable manifold onto the (*ϕ*_C1_, *ϕ*_C2_)-plane will not be the same as the stable manifold of (e*, e*) and the mismatch causes the possible differences in dynamics of the two systems.

### Stability properties of genotype equilibria in class (i) as *r* varies from 0 to 1

The example illustrated in Fig. 4 does not constitute a proof of Proposition 3, but clearly indicates its validity for a particular choice of parameters. Here, we give a detailed analysis for a class of parameters, where we only consider the case *d* > 0 and *b* > *c*; the case *d* < 0 can be obtained in a similar fashion.

For *d* > 0 and *b* > *c*, system (6) has coexisting equilibria (*ϕ*_C1_, *ϕ*_C2_) = (0, 0) and (1, 1) that are both stable in the regime

$$\begin{array}{c} r_{4}^{b}=\frac{c-d}{b+d}<r<\frac{c}{b}=r_{1}^{e}, \end{array}$$

where we used the notation $r_{4}^{b}$ and $r_{1}^{e}$ from Theorem 2. For $r<r_{4}^{b}$ the equilibrium (*ϕ*_C1_, *ϕ*_C2_) = (1, 1) is a source instead of a sink, and for $r>r_{1}^{e}$ the equilibrium (*ϕ*_C1_, *ϕ*_C2_) = (0, 0) is a source instead of a sink. Let us now investigate the stability properties of the corresponding equilibria (0, 0, 0) and (1, 0, 0) of (1).

The stability of equilibria of (1) is determined by the eigenvalues of the Jacobian matrix

$$\begin{array}{ccc} & & \mathrm{Jac}(f_{\mathrm{CC}},f_{\mathrm{CD}},f_{\mathrm{DC}})=\\ & & \left(\begin{array}{ccc} \frac{\partial f_{\mathrm{CC}}\left(w_{\mathrm{CC}}-\bar{w}\right)}{\partial f_{\mathrm{CC}}} & \frac{\partial f_{\mathrm{CC}}\left(w_{\mathrm{CC}}-\bar{w}\right)}{\partial f_{\mathrm{CD}}} & \frac{\partial f_{\mathrm{CC}}\left(w_{\mathrm{CC}}-\bar{w}\right)}{\partial f_{\mathrm{DC}}}\\ \frac{\partial f_{\mathrm{CD}}\left(w_{\mathrm{CD}}-\bar{w}\right)}{\partial f_{\mathrm{CC}}} & \frac{\partial f_{\mathrm{CD}}\left(w_{\mathrm{CD}}-\bar{w}\right)}{\partial f_{\mathrm{CD}}} & \frac{\partial f_{\mathrm{CD}}\left(w_{\mathrm{CD}}-\bar{w}\right)}{\partial f_{\mathrm{DC}}}\\ \frac{\partial f_{\mathrm{DC}}\left(w_{\mathrm{DC}}-\bar{w}\right)}{\partial f_{\mathrm{CC}}} & \frac{\partial f_{\mathrm{DC}}\left(w_{\mathrm{DC}}-\bar{w}\right)}{\partial f_{\mathrm{CD}}} & \frac{\partial f_{\mathrm{DC}}\left(w_{\mathrm{DC}}-\bar{w}\right)}{\partial f_{\mathrm{DC}}} \end{array}\right). \end{array}$$

Note that *f*_CC_ + *f*_CD_ + *f*_DC_ + *f*_DD_ = 1 induces a dependency of *f*_DD_ on all three coordinates. In particular, this means that the partial derivatives must be calculated using the formulation for $\bar{w}=f_{\text{CC}}w_{\text{CC}}+f_{\text{CD}}w_{\text{CD}}+f_{\text{DC}}w_{\text{DC}}+f_{\text{DD}}w_{\text{DD}}$ in terms of *f*_CC_, *f*_CD_ and *f*_DC_ only. The evaluation at an equilibrium simplifies a lot due to the fact that $w_{•}-\bar{w}=0$ for any *f*_•_ ≠ 0.

For the equilibrium (0, 0, 0) almost all terms drop out and we get

$$\begin{array}{ccc} & & \mathrm{Jac}(0,0,0)=\\ & & {\left.\left(\begin{array}{ccc} w_{\mathrm{CC}}-\bar{w} & 0 & 0\\ 0 & w_{\mathrm{CD}}-\bar{w} & 0\\ 0 & 0 & w_{\mathrm{DC}}-\bar{w} \end{array}\right)\right|}_{\left(0,0,0\right)}=\\ & & {\left.\left(\begin{array}{ccc} w_{\mathrm{CC}}-w_{\mathrm{DD}} & 0 & 0\\ 0 & w_{\mathrm{CD}}-w_{\mathrm{DD}} & 0\\ 0 & 0 & w_{\mathrm{DC}}-w_{\mathrm{DD}} \end{array}\right)\right|}_{\left(0,0,0\right)}. \end{array}$$

Hence, the eigenvalues of Jac(0, 0, 0) are on the diagonal and using (2)–(5) with (*f*_CC_, *f*_CD_, *f*_DC_) = (0, 0, 0), we find

$$\begin{array}{ccc} w_{\mathrm{CC}}-w_{\mathrm{DD}} & = & r(b+d)-c,\\ w_{\mathrm{CD}}-w_{\mathrm{DD}} & = & \frac{1}{2}\left(rb-c\right),\\ w_{\mathrm{DC}}-w_{\mathrm{DD}} & = & w_{\mathrm{CD}}-w_{\mathrm{DD}}\\ & = & \frac{1}{2}\left(rb-c\right). \end{array}$$

An equilibrium is stable if and only if all its eigenvalues have negative real part. Since we assume *d* > 0 and *b* > *c*, we find that the stability interval for (0, 0, 0) is

$$\begin{array}{ccc} & & 0<r<\frac{c}{b+d}<\frac{c}{b}\\ & \Leftrightarrow & 0<r<r_{23}^{e}<r_{1}^{e}. \end{array}$$

Note that *E*_23_ merges with (0, 0, 0) when $r=r_{23}^{e}$; this is a transcritical bifurcation that renders two of the three eigenvalues of (0, 0, 0) unstable. The saddle (0, 0, 0) becomes a source at a second transcritical bifurcation when *E*_1_ merges with it at $r=r_{1}^{e}$. We conclude that (0, 0, 0) of (1) destabilises at an *r*-value below the *r*-value at which (0, 0) of (6) destabilises.

Let us now consider the equilibrium (1, 0, 0). The Jacobian matrix becomes

$$\begin{array}{ccc} & & \mathrm{Jac}(1,0,0)=\\ & & {\left.\left(\begin{array}{ccc} \frac{\partial (w_{\mathrm{CC}}-\bar{w})}{\partial f_{\mathrm{CC}}} & \frac{\partial (w_{\mathrm{CC}}-\bar{w})}{\partial f_{\mathrm{CD}}} & \frac{\partial (w_{\mathrm{CC}}-\bar{w})}{\partial f_{\mathrm{DC}}}\\ 0 & w_{\mathrm{CD}}-\bar{w} & 0\\ 0 & 0 & w_{\mathrm{DC}}-\bar{w} \end{array}\right)\right|}_{\left(1,0,0\right)}. \end{array}$$ (15)

Analogous to the case for (0, 0, 0), we have $\bar{w}=w_{\text{CC}}$. Due to the upper triangular structure, the eigenvalues of Jac(1, 0, 0) are also on the diagonal, with the first one determined by

∂(wCC-w¯)∂fCC=∂∂fCC[(1-fCC)(wCC-wDD)-∂∂fCC[fCD(wCD-wDD)-fDC(wDC-wDD)]=-(wCC-wDD).

Here, we used the fact that (*f*_CC_, *f*_CD_, *f*_DC_) = (1, 0, 0). Hence, using (2)–(5), we find that the eigenvalues of (1, 0, 0) are given by

$$\begin{array}{ccc} w_{\mathrm{DD}}-w_{\mathrm{CC}} & = & \left(1-r)b-[r(b+d)-c+(1-r)(b+d)\right]\\ & = & c-d-rb\\ w_{\mathrm{CD}}-w_{\mathrm{CC}} & = & \frac{1}{2}\left[c-r\left(b+2d\right)-\left(1-r\right)d\right]\\ & = & \frac{1}{2}\left[c-d-r\left(b+d\right)\right],\\ w_{\mathrm{DC}}-w_{\mathrm{CC}} & = & w_{\mathrm{CD}}-w_{\mathrm{CC}}\\ & = & \frac{1}{2}\left[c-d-r\left(b+d\right)\right]. \end{array}$$

Therefore, (1, 0, 0) is stable if and only if

$$\begin{array}{ccc} & & r>\frac{c-d}{b}>\frac{c-d}{b+d}\\ & \Leftrightarrow & r>r_{23}^{b}>r_{4}^{b}, \end{array}$$

provided *c* − *d* > 0. For *r* > 0 small enough, (1, 0, 0) is a source; it becomes a saddle with one stable eigenvalue when *r* increases past $r=r_{4}^{b}$, which causes the emergence of *E*_4_ in a transcritical bifurcation. As *r* increases further, (1, 0, 0) becomes stable in a second transcritical bifurcation; this time, two eigenvalues change sign simultaneously (due to the symmetry *f*_CD_ = *f*_DC_ and the bifurcation gives rise to the equilibrium *E*_23_. We conclude that (1, 0, 0) of (1) stabilises at $r=r_{23}^{b}=(c-d)/b$, which lies above the *r*-value $r=r_{4}^{b}=(c-d)/(b+d)$ at which (1, 1) of (6) stabilises.

We can utilise this mismatch in stability intervals to illustrate Proposition 3 for a range of *r*-values with *d* > 0 and *b* > *c*. Consider $r_{23}^{e}<r<r_{1}^{e}$ and let (0, 0) of (6) be the attractor *A*_1_ of Proposition 3. Then almost any initial condition $\left(\phi_{\text{C}1},\phi_{\text{C}2})\in B(A_{1}\right)$ of (6) satisfies the conditions of Proposition 3: almost all initial conditions (*f*_CC_, *f*_CD_, *f*_DC_) of (1) with *f*_CC_ + *f*_CD_ = *ϕ*_C1_ and *f*_CC_ + *f*_DC_ = *ϕ*_C2_ will not converge to (0, 0, 0), because (0, 0, 0) is not stable. (The only exceptions are initial conditions that lie on the one-dimensional stable manifold of the saddle (0, 0, 0).) Similarly, for $r_{4}^{b}<r<r_{23}^{b}$, the equilibrium (1, 1) of (6) is stable, but (1, 0, 0) of (1) is not and Proposition 3 applies.

**Remark 2** *A complete stability analysis of the equilibria of system* (1) *is not included in this paper. As for system* (6), *we believe that the only attractors of system* (1) *are equilibria, but it is far from straightforward to provide a proof. In contrast to the discussion in* [18], *system* (1) *has a three-dimensional phase space and the Poincaré–Bendixson theorem does not apply. Hence, it is possible that an attracting periodic orbit, or even a chaotic attractor exists for system* (1). *However, the planes defined by* {*f*_CC_ = 0}, {*f*_CD_ = 0}, {*f*_DC_ = 0}, *and* {*f*_CC_ + *f*_CD_ + *f*_DC_ = 0} *are all invariant for system* (1), *as is the ‘diagonal’ plane* {*f*_CD_ = *f*_DC_}; *each pair of planes intersects in lines that are also invariant, so that we can use the Poincaré–Bendixson theorem to claim that any such periodic orbit must lie in the interior of one of two tetrahedra, namely, the tetrahedron with vertices* (*f*_CC_, *f*_CD_, *f*_DC_) = (0, 0, 0), (1, 0, 0), (0, 1, 0), *and* $\left(0,\frac{1}{2},\frac{1}{2}\right)$ *or the tetrahedron with vertices* (*f*_CC_, *f*_CD_, *f*_DC_) = (0, 0, 0), (1, 0, 0), $\left(0,\frac{1}{2},\frac{1}{2}\right)$, *and* (0, 0, 1). *System* (1) *is very similar to the class of equivariant vector fields discussed in* [23], *for which all trajectories are attracted by the invariant planes, but the global attractor could be a heteroclinic cycle between three saddles. It may be possible to use the approach in* [23] *for system* (1), *but we leave the complete analysis as a challenge for future research*.

### Analysis of equilibrium states for the genotype model (1)

We complete the analysis of the equilibria for the genotype model (1) in their most general form by providing the proofs of Lemma 1 and Theorem 2.

#### Proof of Lemma 1

Recall that equilibria of (1) satisfy *f*_•_ = 0 or $w_{•}=\bar{w}$. Hence, if all *f*_•_ ≠ 0, we must have $w_{•}=\bar{w}$ for all • ∈ {CC, CD, DC, DD}. This means that

$$\begin{array}{c} \bar{w}=w_{\mathrm{CC}}=w_{\mathrm{CD}}=w_{\mathrm{DC}}=w_{\mathrm{DD}}, \end{array}$$

so we must have equality of all inclusive fitnesses. Equations (2) and (3) give

$$\begin{array}{ccc} & & w_{\mathrm{CC}}=w_{\mathrm{CD}}\\ & \Leftrightarrow & r(b+2d)-c+\\ & & \left(1-r\right)\left[f_{\mathrm{CC}}+f_{\mathrm{CD}}\right]d=0\\ & \Leftrightarrow & f_{\mathrm{CC}}+f_{\mathrm{CD}}=\frac{c-r(b+2d)}{(1-r)d}. \end{array}$$ (16)

Here, we used the assumption *d* ≠ 0. Due to symmetry, even without requiring *f*_CD_ = *f*_DC_, we also have

$$\begin{array}{ccc} & & w_{\mathrm{CC}}=w_{\mathrm{DC}}\\ & \Leftrightarrow & f_{\mathrm{CC}}+f_{\mathrm{DC}}=\frac{c-r(b+2d)}{(1-r)d}. \end{array}$$ (17)

Similarly, (2) and (5) give

$$\begin{array}{ccc} & & w_{\mathrm{CC}}=w_{\mathrm{DD}}\\ & \Leftrightarrow & r(b+d)-c+\\ & & \left(1-r\right)\left[f_{\mathrm{CC}}+\frac{1}{2}f_{\mathrm{CD}}+\frac{1}{2}f_{\mathrm{DC}}\right]d=0 \end{array}$$ (18)

$$\begin{array}{ccc} & \Leftrightarrow & \left[f_{\mathrm{CC}}+\frac{1}{2}f_{\mathrm{CD}}+\frac{1}{2}f_{\mathrm{DC}}\right]=\frac{c-r(b+d)}{(1-r)d}. \end{array}$$ (19)

Using (3) and (5) leads to

$$\begin{array}{ccc} & & w_{\mathrm{DD}}=w_{\mathrm{CD}}\\ & \Leftrightarrow & rb-c+\left(1-r\right)\left[f_{\mathrm{CC}}+f_{\mathrm{DC}}\right]d=0\\ & \Leftrightarrow & f_{\mathrm{CC}}+f_{\mathrm{DC}}=\frac{c-rb}{(1-r)d}. \end{array}$$ (20)

Similarly, (4) and (5) give

$$\begin{array}{ccc} & & w_{\mathrm{DD}}=w_{\mathrm{DC}}\\ & \Leftrightarrow & f_{\mathrm{CC}}+f_{\mathrm{CD}}=\frac{c-rb}{(1-r)d}. \end{array}$$ (21)

Finally, (3) and (4) give

$$\begin{array}{ccc} w_{\mathrm{CD}}=w_{\mathrm{DC}} & \Leftrightarrow & f_{\mathrm{CD}}d=f_{\mathrm{DC}}d\\ & \Leftrightarrow & d=0\;\text{or}\;f_{\mathrm{CD}}=f_{\mathrm{DC}}. \end{array}$$ (22)

It is clear that (16)–(22) can be satisfied simultaneously only if *r* = 0; for example, *w*_CC_ = *w*_CD_ and *w*_DD_ = *w*_DC_ require (16) and (21), that is

$$\begin{array}{ccc} & & \frac{c-r(b+2d)}{(1-r)d}=\frac{c-rb}{(1-r)d}\\ & \Leftrightarrow & \frac{2rd}{(1-r)d}=\frac{2r}{\left(1-r\right)}=0 \end{array}$$

Since 0 < *r* < 1, this proves the Lemma.

#### Proof of Theorem 2

Lemma 1 implies that any equilibrium of (1) must have at least one of its coordinates equal to zero. Furthermore, *f*_CC_ + *f*_CD_ + *f*_DC_ + *f*_DD_ = 1, so the equilibria of (1) can indeed all be classified by the classes listed in Theorem 2. Let us begin with class (i).

*Class (i):*

The equality *f*_CC_ + *f*_CD_ + *f*_DC_ + *f*_DD_ = 1 implies that only (1, 0, 0, 0), (0, 1, 0, 0), (0, 0, 1, 0), and (0, 0, 0, 1) are possible candidates for this class. These four points are equilibria of (1) if the equilibrium condition (11) is satisfied for each of their coordinates. Clearly, we only need to check (11) for the single nonzero coordinate *f*_•_ = 1, for which we require $w_{•}=\bar{w}$. However, the mean fitness,

$$\begin{array}{c} \bar{w}=f_{\mathrm{CC}}w_{\mathrm{CC}}+f_{\mathrm{CD}}w_{\mathrm{CD}}+f_{\mathrm{DC}}w_{\mathrm{DC}}+f_{\mathrm{DD}}w_{\mathrm{DD}}, \end{array}$$

simply reduces to *w*_•_ if three of the four frequencies are zero. Hence, (1, 0, 0, 0), (0, 1, 0, 0), (0, 0, 1, 0), and (0, 0, 0, 1) are all equilibria and there are no restrictions on *r* for their existence.

*Class (ii):*

This class contains all equilibria with two coordinates equal to zero. Suppose *f*_CC_ = 0 and consider the case *f*_CD_ = 0, while *f*_DC_, *f*_DD_ ≠ 0. Then (21) must hold in order to satisfy (11), but *f*_CC_ + *f*_CD_ = 0, so there is no (generic) solution. Similarly, if we assume *f*_CD_ ≠ 0 and *f*_DC_ = 0, then (20) implies

$$\begin{array}{c} \frac{c-rb}{(1-r)d}=0\Leftrightarrow r=\frac{c}{b}, \end{array}$$

which is not generic. At the special value $r=\frac{c}{b}$ a two-dimensional continuum of equilibria (0, 0, *f*_DC_, *f*_DD_) and another two-dimensional continuum of equilibria (0, *f*_CD_, 0, *f*_DD_) exist that are both not persistent under variations in *r*. Hence, a generic equilibrium from class (ii) with *f*_CC_ = 0 must have *f*_DD_ = 0. Then (22) holds, which gives the candidate $\left(0,\frac{1}{2},\frac{1}{2},0\right)$. Since *w*_DC_ = *w*_CD_, the mean fitness becomes

$$\begin{array}{c} \bar{w}=\frac{1}{2}w_{\mathrm{CD}}+\frac{1}{2}w_{\mathrm{DC}}=w_{\mathrm{CD}}=w_{\mathrm{DC}}, \end{array}$$

so $\left(0,\frac{1}{2},\frac{1}{2},0\right)$ is indeed an equilibrium. Note that this equilibrium exists without restrictions on *r*.

The only other option for equilibria in this class are equilibria with two zero coordinates and *f*_CC_ ≠ 0. If we assume that the other nonzero coordinate is *f*_CD_ ≠ 0, then (16) implies

$$\begin{array}{c} \frac{c-r(b+2d)}{(1-r)d}=1\Leftrightarrow r=\frac{c-d}{b+d}, \end{array}$$

because *f*_DC_ = *f*_DD_ = 0, so that *f*_CC_ + *f*_CD_ = 1. This is again not generic. The same applies to the case *f*_CD_ = *f*_DD_ = 0, using (17), and the only remaining candidate is an equilibrium with *f*_CD_ = *f*_DC_ = 0. For this case (19) applies and we find

$$\begin{array}{c} f_{\mathrm{CC}}=\frac{c-r(b+d)}{(1-r)d}. \end{array}$$

The value for *f*_DD_ follows from the remainder *f*_DD_ = 1 − *f*_CC_. The equality of the inclusive fitnesses for all nonzero frequencies again implies $\bar{w}=w_{\text{CC}}=w_{\text{DD}}$. Hence, the candidate *E*_23_ as given in (12) is indeed an equilibrium.

The existence interval of *E*_23_ is determined by the fact that all coordinates of *E*_23_ must lie in the interval [0, 1]; it suffices to check this for the *f*_CC_-coordinate of *E*_23_, since *f*_CC_ + *f*_DD_ = 1 then implies 0 ≤ *f*_DD_ ≤ 1 as well. Let us first consider the case with *d* > 0; we have:

$$\begin{array}{ccc} & & 0\leq \frac{c-r(b+d)}{(1-r)d}\leq 1\\ & \Leftrightarrow & \left\{\begin{array}{cccc} c-r(b+d) & \geq & 0 & \text{and}\\ c-r(b+d) & \leq & (1-r)d, \end{array}\right.\\ & \Leftrightarrow & \left\{\begin{array}{cccc} r & \leq & \frac{c}{b+d} & \text{and}\\ r & \geq & \frac{c-d}{b}. \end{array}\right. \end{array}$$

Hence, the existence interval is $\frac{c-d}{b}\leq r\leq \frac{c}{b+d}$, which only makes sense if

$$\begin{array}{c} \frac{c-d}{b}<\frac{c}{b+d}\Leftrightarrow c-b<d. \end{array}$$ (23)

The bounds $r_{23}^{b}$ and $r_{23}^{e}$ defined in Theorem 2 take into account that one could have *c* − *d* < 0, in which case $0<r<\frac{c}{b+d}$.

The case *d* < 0 is analogous, with ‘≤’ replaced by ‘≥’ and vice versa as soon as the inequality is multiplied by (1 − *r*)*d*. Note that we must consider the possibility *b* + *d* < 0, but this leads to *r* < 0, which is not acceptable. Hence, we have *b* + *d* > 0 and the bounds $r_{23}^{b}$ and $r_{23}^{e}$ simply swap places. We now need $r_{23}^{e}<r_{23}^{b}$, which leads to the same condition *c* − *b* < *d* as derived in (23) for *d* > 0; note that *c* − *b* < *d* ⇒ *b* + *d* > 0.

*Class (iii):*

The final class consists of equilibria with three nonzero coordinates. We obtain *E*_1_ given by (13) if we assume *f*_CC_ = 0. Indeed, for this case, (20), (21) and (22) must hold, which requires

$$\begin{array}{c} f_{\mathrm{CD}}=f_{\mathrm{DC}}=\frac{c-rb}{(1-r)d}, \end{array}$$

and the value for *f*_DD_ follows from the fact that all frequencies sum up to one. As before, the equality *w*_CD_ = *w*_DC_ = *w*_DD_ implies that $\bar{w}$ is equal to each of these inclusive fitnesses and *E*_1_ is, indeed, an equilibrium.

The existence interval of *E*_1_ is then determined by the values of *r* for which $f_{\text{CD}}=f_{\text{DC}}\in [0,\frac{1}{2}]$; this automatically implies *f*_DD_ ∈ [0, 1]. Let us first consider the case *d* > 0. If we assume 2*b* − *d* > 0 then we have:

$$\begin{array}{ccc} & & 0\leq \frac{c-rb}{(1-r)d}\leq \frac{1}{2}\\ & \Leftrightarrow & \left\{\begin{array}{cccc} c-rb & \geq & 0 & \text{and}\\ 2(c-rb) & \leq & (1-r)d, \end{array}\right.\\ & \Leftrightarrow & \left\{\begin{array}{cccc} r & \leq & \frac{c}{b} & \text{and}\\ r & \geq & \frac{2c-d}{2b-d}. \end{array}\right. \end{array}$$

These bounds lead to an *r*-interval if $\frac{2c-d}{2b-d}<\frac{c}{b}$, which holds if *b* > *c*; note that the additional condition 0 < *r* < 1 defines the bounds $r_{1}^{b}$ and $r_{1}^{e}$ given in Theorem 2. If *d* is large and 2*b* − *d* < 0 then *E*_1_ exists for $0<r<\frac{c}{b}$, if *b* > *c*, and for $0<r<\frac{2c-d}{2b-d}$, if *b* < *c*.

The case *d* < 0 is again analogous, and we get $r_{1}^{e}<r<r_{1}^{b}$. The condition $r_{1}^{e}<r_{1}^{b}$ leads to the requirement *b* > *c*, which automatically ensures that this *r*-interval is contained in [0, 1].

Let us now consider the possible existence of an equilibrium with *f*_CD_ = 0 and all other coordinates nonzero. This means that (17), (19) and (21) must hold. Since *f*_CD_ = 0, equation (21) defines *f*_CC_, and combined with (17), this gives

$$\begin{array}{ccc} f_{\mathrm{DC}} & = & \frac{c-r(b+2d)}{(1-r)d}-f_{\mathrm{CC}}\\ & = & \frac{c-r(b+2d)}{(1-r)d}-\frac{c-rb}{(1-r)d}\\ & = & \frac{-2dr}{(1-r)d}=\frac{-2r}{\left(1-r\right)}<0; \end{array}$$

here, we used the fact that 0 < *r* < 1. Hence, there is no admissible equilibrium in class (iii) that satisfies *f*_CD_ = 0. A similar argument holds for the case with *f*_DC_ = 0.

The only other possibility is an equilibrium with all nonzero coordinates except for *f*_DD_ = 0. We must satisfy (16), (17) and (22), which implies

$$\begin{array}{ccc} f_{\mathrm{CC}}+f_{\mathrm{CD}} & = & f_{\mathrm{CC}}+f_{\mathrm{DC}}\\ & = & \frac{c-r(b+2d)}{(1-r)d}. \end{array}$$

Furthermore, *f*_CC_ + *f*_CD_ + *f*_DC_ = 1, so

$$\begin{array}{ccc} f_{\mathrm{CD}} & = & f_{\mathrm{DC}}\\ & = & 1-\frac{c-r(b+2d)}{(1-r)d}\\ & = & \frac{d-c+r(b+d)}{(1-r)d}, \end{array}$$

which fixes *f*_CC_ as well. Hence, *E*_4_ as defined in (14) is an equilibrium of system (1).

As before, we find the existence interval of the equilibrium *E*_4_ using the condition $f_{\text{CD}}=f_{\text{DC}}\in [0,\frac{1}{2}]$. Let us first consider the case *d* > 0, which leads to:

$$\begin{array}{ccc} & & 0\leq \frac{d-c+r(b+d)}{(1-r)d}\leq \frac{1}{2}\\ & \Leftrightarrow & \left\{\begin{array}{cccc} d-c+r(b+d) & \geq & 0 & \text{and}\\ 2(d-c+r(b+d)) & \leq & (1-r)d, \end{array}\right.\\ & \Leftrightarrow & \left\{\begin{array}{cccc} r & \geq & \frac{c-d}{b+d} & \text{and}\\ r & \leq & \frac{2c-d}{2b+3d}. \end{array}\right. \end{array}$$

As for the other equilibria, we must show that these bounds lead to a nontrivial *r*-interval. We have

$$\begin{array}{c} \frac{c-d}{b+d}<\frac{2c-d}{2b+3d}\Leftrightarrow \frac{1}{2}\left(c-b\right)<d, \end{array}$$ (24)

so *E*_4_ can only exist for *d* > 0 if $\frac{1}{2}(c-b)<d$; the bounds $r_{4}^{b}$ and $r_{4}^{e}$ defined in Theorem 2 take into account that 0 < *r* < 1 as well.

For the case *d* < 0 we have (1 − *r*)*d* < 0 and we find the existence interval $r_{4}^{e}<r<r_{4}^{b}$, provided the same bound $\frac{1}{2}(c-b)<d$ from (24) is satisfied; here we assume *b* + *d* > 0 and 2*b* + 3*d* > 0. The case *b* + *d* < 0 leads to an interval with *r* < 0, which is not admissible; the case *b* + *d* > 0, but 2*b* + 3*d* < 0 also requires *r* < 0. Note that the condition $\frac{1}{2}(c-b)<d$ implies

$$\begin{array}{ccc} b+d & > & b+\frac{1}{2}\left(c-b\right)\\ & = & \frac{1}{2}\left(c+b\right)>0, \end{array}$$

and

$$\begin{array}{ccc} 2b+3d & > & 2b+\frac{3}{2}\left(c-b\right)\\ & = & \frac{1}{2}\left(3c+b\right)>0. \end{array}$$

This concludes the investigation of all possible equilibria for system (1). In total, we found the eight equilibria listed in Theorem 2 and there are no other equilibria.

Note that $\frac{2c-d}{2b+3d}<\frac{2c-d}{2b-d}$ if *d* > 0 and these values are positive, which means that *E*_1_ and *E*_4_ do not both exist at the same time. Similarly, the opposite equality is satisfied for *d* < 0, provided these values are positive, so that the same conclusion holds. The equilibrium *E*_23_ can coexist with either *E*_1_ or *E*_4_ in certain regions of parameter space. Therefore, at most seven equilibria coexist.

## Discussion

For a simple yet general evolutionary game theory model of social evolution, in which behaviour is conditioned on social role occupied, recent analysis has shown that the stable equilibria under selection are the same regardless of whether one considers selection acting on the entire strategy [2, 6], or acting on independent ‘genes’ for each role [9]. Thus, it may be tempting to assume that, for certain kinds of sufficiently simple models, strategy-level and independent-gene-level approaches yield equivalent answers. Here, we have presented an analysis using the tools of dynamical systems theory, for the simple case of asymmetric non-additive donation games played between relatives. Although we present our analyses in terms of this game, the payoff matrix we use is equivalent to a fully general 2-player payoff matrix [9]. This analysis reveals the following main points: first, the gene dynamics and the genotype dynamics cannot be made topologically equivalent in a dynamical systems sense, since the dimensions of the respective phase spaces are different. It is also not possible to ‘slave’ the dynamics of the higher-dimensional gene dynamics model to the genotype model, because the two models differ in their number of equilibria and in the locations of some of these equilibria. Second, we find additional equilibria for the genotype model to those previously found using techniques from evolutionary game theory, since we find unstable equilibria as well as the previously-discovered stable equilibria. Third, by observing that the unstable equilibria under the gene and the genotype dynamics are different, we show that although the stable equilibria are the same in the two systems, initial conditions often exist in which the population equilibria that result under selection in each system are different. That is, for the same starting population the two different model analyses predict different evolutionary outcomes.

Our results provide an interesting contrast to earlier results from the population genetics literature, that the genetic bases of traits under selection affect whether population equilibria will maximise population mean fitness. These population genetics approaches, briefly reviewed in [24], rest on analysis of sexual models. In particular, analysis of population genetics models shows that the concept of an ‘adaptive landscape’ independent of genetic details is incorrect; while a single-locus model may predict that population equilibria maximise population-mean-fitness, moving to a two-locus model can break the correspondence between equilibria under selection and fitness maxima [10]. Our analysis is an evolutionary game theory one, which is inherently asexual; strategies, or strategy components (‘genes’) reproduce directly. Our analysis of the particular social game presented here also demonstrates a different effect, since here the stable equilibria are the same and correspond with fitness-maximising equilibria, but the selected equilibria can differ. The fact that it is only equilibrium selection, rather than the stable equilibria themselves, that is affected by using the analytically simpler model of this general game may be of interest to some researchers. For applications in which the only result of interest is the stable equilibria under selection, using the simpler two-dimensional systems of equations is safe; for applications in which equilibrium selection does matter, our results describe the areas of disagreement between the predictions of the two-dimensional and three-dimensional representations of selection, enabling an informed choice to be made about which is appropriate to use. The fact that, as described above, the game analysed is actually equivalent to a fully general game matrix for 2-player interactions where players occupy distinct behavioural roles should reinforce this potential interest.

## Supporting Information

S1 Appendix Topological non-equivalence of the two models. One may be tempted to believe that the higher-dimensional system (1) implies the behaviour of system (6), because *ϕ*_C1_ and *ϕ*_C2_ should evolve in the same way as *f*_CC_ + *f*_CD_ and *f*_CC_ + *f*_DC_, respectively. While it is straightforward to show that the two systems are not topologically equivalent, also not in this sense, the proof is rather tedious and not very insightful. Therefore, we provide this proof in the Supporting Information for completeness.
(PDF)
