# Supplementary material for: Adaptive Topographies and Equilibrium Selection in an Evolutionary Game
Source: PLoS One. 2015 Feb 23;10(2):e0116307. doi: 10.1371/journal.pone.0116307 (PMC4338017; doi:10.1371/journal.pone.0116307)
Supplement: S1 Appendix — One may be tempted to believe that the higher-dimensional system (1) implies the behaviour of system (6), because ϕ C1 and ϕ C2 should evolve in the same way as f CC + f CD and f CC + f DC, respectively. While it is straightforward to show that the two systems are not topologically equivalent, also not in this sense, the proof is rather tedious and not very insightful. Therefore, we provide this proof in the Supporting Information for completeness. (PDF) [file pone.0116307.s001.pdf]

S1 APPENDIX: ADAPTIVE TOPOGRAPHIES AND EQUILIBRIUM SELECTION IN AN EVOLUTIONARY GAME

HINKE M. OSINGA AND JAMES A. R. MARSHALL

TOPOLOGICAL NON-EQUIVALENCE OF THE TWO MODELS

Note that the relationships $\phi_{\mathrm{C1}} + \phi_{\mathrm{D1}} = 1$ and $\phi_{\mathrm{C2}} + \phi_{\mathrm{D2}} = 1$ for system (6) mean that we only need to consider the cases $\dot{\phi}_{\mathrm{Ci}}$ with $i \in \{1, 2\}$. We show here that $\dot{\phi}_{\mathrm{C1}} \neq \dot{f}_{\mathrm{CC}} + \dot{f}_{\mathrm{CD}}$, where $\dot{f}_{\mathrm{CC}}$ and $\dot{f}_{\mathrm{CD}}$ are given by equation (1); the case for $\dot{\phi}_{\mathrm{C2}}$ is similar.

Let us consider the equation for $\dot{\phi}_{\mathrm{C1}}$. We are given

$$\dot{\phi}_{\mathrm{C1}} = \phi_{\mathrm{C1}}\ (1 - \phi_{\mathrm{C1}})\ (\omega_{\mathrm{C1}} - \omega_{\mathrm{D1}}),$$

where

$$\omega_{\mathrm{C1}} - \omega_{\mathrm{D1}} = r\,b - c + (1 + r)\,d\,\phi_{\mathrm{C2}}.$$

We have a similar equation for $\dot{\phi}_{\mathrm{C2}}$, but let us assume that $\phi_{\mathrm{C2}} = \phi_{\mathrm{C1}}$, that is, we consider the diagonal dynamics only, as given by the single equation

$$\dot{\phi} = \phi\ (1 - \phi)\ [r\,b - c + (1 + r)\,d\,\phi] := \phi\ (1 - \phi)\ G(\phi). \tag{1}$$

Since $\phi_{\mathrm{C1}} = f_{\mathrm{CC}} + f_{\mathrm{CD}}$ and $\phi_{\mathrm{C2}} = f_{\mathrm{CC}} + f_{\mathrm{DC}}$, the equality $\phi_{\mathrm{C2}} = \phi_{\mathrm{C1}}$ implies $f_{\mathrm{DC}} = f_{\mathrm{CD}}$. Under this assumption, the mean fitness becomes

$$\overline{\omega} = (r\,b - c + [1 - r]\,b + [1 - r]\,d\,[f_{\mathrm{CC}} + f_{\mathrm{CD}}])\ [f_{\mathrm{CC}} + f_{\mathrm{CD}}] + r\,d\,f_{\mathrm{CC}}.$$

Hence, we have

$$\begin{aligned} \dot{f}_{\mathrm{CC}} &= f_{\mathrm{CC}}\ (w_{\mathrm{CC}} - \overline{\omega}) \\ &= f_{\mathrm{CC}}\ \{(1 - [f_{\mathrm{CC}} + f_{\mathrm{CD}}])\,(r\,b - c + [1 - r]\,d\,[f_{\mathrm{CC}} + f_{\mathrm{CD}}]) \\ &\qquad + r\,d\,(1 - f_{\mathrm{CC}})\} \end{aligned} \tag{2}$$

and

$$\begin{aligned} \dot{f}_{\mathrm{CD}} &= f_{\mathrm{CD}}\ (w_{\mathrm{CD}} - \overline{\omega}) \\ &= \tfrac{1}{2}\,f_{\mathrm{CD}}\ \{(1 - 2\,[f_{\mathrm{CC}} + f_{\mathrm{CD}}])\,(r\,b - c + [1 - r]\,d\,[f_{\mathrm{CC}} + f_{\mathrm{CD}}]) \\ &\qquad - 2\,r\,d\,f_{\mathrm{CC}}\} \end{aligned} \tag{3}$$

If we write $g = f_{\mathrm{CC}} + f_{\mathrm{CD}}$, then (2) and (3) give

$$\begin{aligned} \dot{g} &= \dot{f}_{\mathrm{CC}} + \dot{f}_{\mathrm{CD}} \\ &= g\,(1 - g)\,(r\,b - c + [1 - r]\,d\,g) \\ &\qquad + r\,d\,(1 - f_{\mathrm{CC}})\,g - \tfrac{1}{2}\,f_{\mathrm{CD}}\,(r\,b - c + [1 - r]\,d\,g) - r\,d\,f_{\mathrm{CD}} \\ &= g\,(1 - g)\,G(g) - \tfrac{1}{2}\,G(g)\,f_{\mathrm{CD}} - r\,d\,[g\,(1 - g) + 2\,g\,f_{\mathrm{CC}} + f_{\mathrm{CD}}]. \end{aligned}$$

Comparing with (1), we observe that already the diagonal dynamics does not satisfy $\dot{\phi}_{\mathrm{C1}} = \dot{f}_{\mathrm{CC}} + \dot{f}_{\mathrm{CD}}$.

□
